# Dietary Inflammatory Index Is Associated With Inflammation in Japanese Men

**DOI:** 10.3389/fnut.2021.604296

**Published:** 2021-04-09

**Authors:** Ayaka Kotemori, Norie Sawada, Motoki Iwasaki, Taiki Yamaji, Nitin Shivappa, James R. Hebert, Junko Ishihara, Manami Inoue, Shoichiro Tsugane

**Affiliations:** ^1^Epidemiology and Prevention Group, Center for Public Health Sciences, National Cancer Center, Tokyo, Japan; ^2^Department of Food and Life Science, Azabu University, Kanagawa, Japan; ^3^Department of Epidemiology and Biostatistics, Arnold School of Public Health, University of South Carolina, Columbia, SC, United States; ^4^Cancer Prevention and Control Program, University of South Carolina, Columbia, SC, United States; ^5^Department of Nutrition Connecting Health Innovations Limited Liability Company, Columbia, SC, United States

**Keywords:** dietary inflammatory index, food frequency questionnaire, inflammatory biomarker, high-sensitivity C-reactive protein, Japanese

## Abstract

**Background:** Dietary components are known to affect chronic low-grade inflammation status. The dietary inflammatory index (DII®) was developed to measure the potential impact of a diet on an individual's inflammatory status, and it has been validated mainly in Western countries.

**Objective:** This study aimed to examine the validity of the energy-adjusted DII (E-DII^TM^) using high-sensitivity C-reactive protein (hs-CRP) concentration in Japanese men and women.

**Methods:** In total, 6,474 volunteers from a cancer-screening program (3,825 men and 2,649 women) completed a food frequency questionnaire (FFQ) and their hs-CRP concentrations were evaluated. E-DII scores were calculated on the basis of 30 food parameters derived from the FFQ. Higher E-DII scores reflect a greater pro-inflammatory potential of the diet. The associations between E-DII quartiles and hs-CRP concentration were assessed using regression models adjusted for age, body mass index, smoking status, and amount of physical activity.

**Results:** Mean E-DII in men and women was + 0.62 ± 1.93 and −1.01 ± 2.25, respectively. The proportion of men and women who had hs-CRP concentration >3 mg/L was 4.7 and 3.1%, respectively. A significant positive association was observed between E-DII score and hs-CRP concentration in men; geometric mean of hs-CRP concentration in the lowest and highest E-DII quartiles was 0.56 mg/L and 0.67 mg/L (*P*_trend_ < 0.01), respectively. The odds ratio (95% confidence interval) of having an elevated hs-CRP concentration (>3 mg/L) was 1.72 (1.10–2.67) in the highest E-DII quartile (*P*_trend_ = 0.03) in men. However, no association was observed between E-DII score and hs-CRP concentration in women, except in those not taking prescription medications.

**Conclusions:** DII was associated with inflammation status in Japanese men, but the association was limited in Japanese women.

## Introduction

Low-grade chronic inflammation promotes the development of lifestyle-related chronic diseases such as cancer (1–3), cardiovascular disease (4), diabetes (5), and depression (6). High-sensitivity C-reactive protein (hs-CRP) is a well-known inflammatory biomarker, and previous studies have reported that elevated concentrations of hs-CRP are associated with an increased risk of cancer and the incidence of other chronic diseases (1–3, 7, 8).

Dietary components are one of the key factors affecting an individual's inflammatory status (9). High intake of dietary fiber has been shown to be associated with low hs-CRP concentrations, whereas high intake of saturated fatty acids leads to elevation in hs-CRP concentration (10). In addition, a healthy dietary pattern, characterized by high intake of fruits, vegetables, and fish, has been associated with lower hs-CRP concentrations (11). Mediterranean diet also has been shown to reduce hs-CRP concentrations in randomized controlled trials (12, 13). In contrast, Western dietary patterns, characterized by high intake of red and processed meat, is associated with high hs-CRP concentrations (11). These reports suggest that dietary components and dietary patterns may have a contrasting effect on inflammatory status. Therefore, a comprehensive index is required to understand the potential impact of whole diet on inflammatory status.

The Dietary Inflammatory Index (DII®) is a literature-based dietary score that was developed to measure the potential impact of a diet on the inflammatory status of an individual; a high DII score reflects pro-inflammatory potential of the diet, whereas a low DII score reflects the anti-inflammatory potential of the diet (14, 15). To date, 30 validation studies have been performed between DII and various inflammatory markers, mainly in Western countries (15–18). In addition, the DII has also been shown to be associated with an increased risk of many chronic diseases including cancer (19–21). In Japan, one study reported that a higher DII score increased the risk of upper aerodigestive tract cancers (22). Among the 30 construct validations performed throughout the world, two have been performed in Japan (18, 23), the results of which are inconsistent with regard to sex-specific analyses. Dietary habits and inflammatory status are considerably different between Japanese and Western populations (23–26). Therefore, it is important to determine the utility of the DII to quantify the inflammatory potential of diet in Japanese men and women.

Previously, we conducted a cross-sectional validation study in a subsample from the Japan Public Health Center-based prospective (JPHC) study but could not identify a positive association between the DII and inflammatory status in women (23). The underlying reasons for the null result could be the small sample size of the study and the fact that women consume a more anti-inflammatory diet than do men (23). Therefore, the present study aimed to assess the associations between DII scores and hs-CRP concentrations in a large number of Japanese men and women.

## Methods

### Study Design and Participants

The National Cancer Center of Japan started a cancer-screening program in February 2004. The total number of participants in the present study was 7,919 healthy volunteers (4,664 men and 3,255 women) aged 40–69 years who had participated in the cancer-screening program from May 2009 to December 2013. The blood samples of participants were collected during cancer screening. They also answered self-administered questionnaires for demographic, lifestyle, and dietary information. This study was approved by the Institutional Review Board of the National Cancer Center, Tokyo, Japan (approval number G15-01 and 2016-165). The study aims and protocols were explained to all participants, and each participant provided written informed consent before enrolment in the study.

### Self-Administered Questionnaire

The self-administered questionnaire was designed to obtain participants' demographic, lifestyle, and dietary information, including information on height and weight at the time of examination, smoking status, physical activity, and past medical history of cancer, stroke, and myocardial infarction. Dietary information was obtained using a validated food frequency questionnaire (FFQ), which had questions on the consumption of 188 food and beverage items other than supplements. Energy and nutrient intakes were calculated by taking the sum of the products of eating frequency, portion size, and energy and nutrient content of each food, while referring to the Standard Tables of Food Composition in Japan, Fifth Revised and Enlarged Edition (27). A validation study for the energy and nutrient intake had already been conducted in a subsample of the examinees of the cancer screening program, wherein FFQ-derived data were compared with the data derived from four-day weighed dietary records. The correlation coefficients of energy intake in men and women were 0.53 and 0.34, respectively, and the median correlation coefficients of 45 nutrients were 0.57 and 0.47, respectively. More detailed information has been described elsewhere (28).

### Calculation of DII Score

The DII is a literature-based dietary index calculated from 45 nutrients and food components to assess the potential impact of a diet on the inflammation status; a high score indicates pro-inflammatory potential of the diet and a low score indicates anti-inflammatory potential of the diet. The index was developed by reviewing and scoring 1,943 peer-reviewed publications, which included cell culture experiments, animal experiments, and human studies, and examined the associations between various dietary components and six inflammatory biomarkers [interleukin (IL)-1β, IL-4, IL-6, IL-10, Tumor Necrosis Factor α, and CRP] (14). Inflammatory effect scores for each of the DII components were determined by considering the direction of the effect on inflammation, weight of study design, and number of publications. The inflammatory effect scores are available from the publication (14). To calculate an individual's DII score, dietary intake of the DII components was standardized as a Z-score using global daily mean intake and converted into proportion scores in the study population. The global daily mean intake was calculated from a national database of dietary intake in eleven countries including Japan (14). Then, the individual's DII score was calculated as the sum of the products of the centered proportion score and the inflammatory effect score for each of the DII components.

In the present study, we computed the energy-adjusted DII (E-DII^TM^) using the energy-adjusted intake of the following 30 DII components (out of 45 possible components) (23): protein, total fat, saturated fatty acid, monounsaturated fatty acid, polyunsaturated fatty acid, n-3 fatty acid, n-6 fatty acid, cholesterol, carbohydrate, magnesium, iron, zinc, retinol equivalent, beta-carotene, vitamin D, alpha-tocopherol, vitamin B_1_, vitamin B_2_, niacin, vitamin B_6_, vitamin B_12_, folate, vitamin C, total dietary fiber, isoflavone, ethanol, onion, green or black tea, and caffeine. Some of the components (such as thyme or oregano and rosemary) are not commonly consumed by the Japanese (23). Energy adjustment was done using the density method, and the amount per 1,000 kcal was used for E-DII calculation.

### Blood Sampling and Measurement of Inflammatory Biomarkers

Fasting blood samples were collected along with the self-administered questionnaire data before any cancer screening procedures on the first day of screening. Venous blood was drawn into a vacutainer tube without anticoagulant, and the samples were centrifuged to obtain serum.

Serum hs-CRP concentrations were measured using a commercial reagent kit (Nanopia CRP, Sekisui Medical Co., Ltd., Tokyo, Japan) with an automated analyzer JCA-BM6070 (Jeol Ltd., Tokyo, Japan) at National Cancer Center, Tokyo, Japan. The intra-assay coefficient of variation was 1.8% for 1 mg/L, 1.3% for 2 mg/L, and 0.7% for 82 mg/L of CRP concentration (*n* = 20 each) according to the manufacturer's data (29). The detection range of the kit was 0.2 mg/L−420 mg/L. In case the values were lower than the lower limit of quantification, we assigned a value of 0.1 mg/L.

### Statistical Analysis

Among the 7,919 participants, 1,445 were excluded on the basis of following criteria: BMI < 14 or >40 kg/m^2^ (*n* = 13); missing data on smoking status (*n* = 1); missing data on metabolic equivalents (*n* = 1); participants with a history of any cancer, stroke, and myocardial infarction (*n* = 877); extreme energy intake identified in the upper and lower 2.5%-tile (*n* = 349); missing data on alcohol consumption (*n* = 120); and missing data on hs-CRP or hs-CRP concentration >10 mg/L (*n* = 84). Therefore, data from 6,474 participants (3,825 men and 2,649 women) were included in the statistical analysis. Cohen's effect size *f* ^2^ and noncentral *F* distribution *F*(*df*_*Reg*_, *df*_*Res*_, λ) were used to calculate sample size *n* for multiple regression analysis; where $f^{2}=\frac{R^{2}}{1-R^{2}}$, *R*^2^ is the coefficient of determination, *df*_*Reg*_ is the degree of freedom for regression (= k), *df*_*Res*_ is the degree of freedom for residual (= n – k – 1), and the noncentral parameter λ is λ = *f*^2^*n* (30). When we set the effect size = 0.01, k = 12, power = 0.8, and significance level = 0.05, the sample size *n* was calculated to be 1,745.

Participants' characteristics were summarized using percentages for categorical variables; mean and standard deviation (SD), as well as median and interquartile range for continuous variables; and geometric mean and coefficient of variation (CV) for log-transformed hs-CRP. The CV was also calculated with the log-transformed value using the formula: CV = (e^SD^−1)^1/2^ (31). Comparison of characteristics between men and women was done using the chi-square test for categorical variables. The Mann–Whitney U test was used for all continuous variables, as the Kolmogorov–Smirnov test revealed non-normal distribution of all variables except men's height.

Multiple regression analyses were performed for the associations between the E-DII score and food group intake. Associations between the E-DII scores and hs-CRP concentration were evaluated using multivariable linear regression models. Linear trends across E-DII quartiles were calculated using natural log-transformed hs-CRP concentration as a dependent variable. To interpret a partial regression coefficient estimated using this model, e (Euler's number) should be exponentiated with that coefficient. The models were adjusted for age (years; continuous), BMI (kg/m^2^; continuous), smoking status (current, past, and never), regular prescription medicine use (yes or no), and daily total physical activity level (MET-h/d; continuous). Sensitivity analyses were performed stratified by age, BMI, and regular prescription medicine use. In addition, logistic regression models were applied to estimate the odds ratio (OR) of having elevated hs-CRP concentration (>3 mg/L) across quartiles of E-DII score because this is a clinically relevant cut-off point for chronic inflammation status (32). All statistical analyses were performed using Statistical Analysis Systems software, version 9.3 (SAS Institute Inc., Cary, NC, USA), and the significance level was set at *p* < 0.05.

## Results

### Characteristics of the Participants

Table 1 shows the characteristics of the participants stratified by sex. BMI and the proportion of smokers and regular prescription medicine users were significantly higher in men than in women. The amount of physical activity was significantly higher in women than in men. The E-DII score was significantly higher in men (+0.62 ± 1.93) than in women (−1.01 ± 2.25) (*p* < 0.001). Hs-CRP concentrations also were significantly higher in men than in women; mean (CV) was 0.58 mg/L (1.21) and 0.44 mg/L (1.24), respectively (*p* < 0.001). The proportion of participants who had hs-CRP concentration >3 mg/L was also significantly higher in men than in women (4.7 and 3.1%, respectively; *p* < 0.001). The comparisons between the prescription medication non-users and users are shown in Supplementary Table 1. In both sexes, users were older and had higher BMI, lower DII, and higher hs-CRP concentrations than non-users did.

**Table 1:** Characteristics of the volunteers of the cancer-screening program from May 2009 to December 2013 at National Cancer Center Japan.

	**Men**		**Women**		
	**(*n* = 3825)**		**(*n* = 2649)**		***p*-value**
	**Mean (SD)**	**Median (interquartile range)**	**Mean (SD)**	**Median (interquartile range)**	
Age (years)	56.7 ± 8.3	58 (50, 64)	56.5 ± 8.2	58 (50, 64)	0.45
Height (cm)	169.5 ± 5.9	169.6 (165.5, 173.4)	156.4 ± 5.5	156.4 (152.5, 160.0)	<0.001
Weight (kg)	68.6 ± 9.5	67.6 (62.2, 74.2)	53.7 ± 8.1	52.7 (48.5, 57.8)	<0.001
BMI (kg/m^2^)	23.9 ± 2.9	23.7 (21.9, 25.5)	21.9 ± 3.1	21.5 (19.9, 23.5)	<0.001
**Smoking status (%)**					
Current	15.4		5.5		<0.001
Former	52.1		15.2		
Never	32.4		79.3		
Regular prescription medicine user (%)	48.1		44.7		0.008
Physical activity (MET-h/d)	36.6 ± 3.2	35.9 (34.6, 37.6)	38.0 ± 3.7	37.1 (35.6, 39.3)	<0.001
E-DII (/1000 kcal)^a^	0.62 ± 1.93	0.88 (−0.62, 2.07)	−1.01 ± 2.25	−1.00 (−2.71, 0.63)	<0.001
Crude hs-CRP (mg/L)	0.91 ± 1.14	0.5 (0.3, 1.0)	0.72 ± 1.04	0.4 (0.2, 0.8)	<0.001
hs-CRP (mg/L)^b^	0.58 ± 1.21	—	0.44 ± 1.24	—	<0.001
>3 mg/L of hs-CRP (%)	4.7		3.1		<0.001

*Chi-square test and Mann–Whitney U test are used for statistical analyses*.

a *E-DII is calculated from dietary intake converted per 1000 kcal*.

b *Geometric mean and coefficient of variation were presented for log-transformed inflammatory biomarkers. Coefficient of variation is calculated using the formula: CV = (e^SD^−1)^1/2^*.

*BMI, body mass index; E-DII, energy-adjusted dietary inflammatory index; hs-CRP, high-sensitive C-reactive protein; MET, metabolic equivalent*.

### Associations Between Food Group Intake and E-DII Score

Table 2 shows the results of multiple regression analyses between E-DII score and food group intake, stratified by sex. The findings suggest that a higher E-DII score was associated with a higher intake of sugar, meat, and confectioneries in men, while a higher E-DII score was associated only with higher sugar intake in women.

**Table 2:** Standardized beta coefficient with 95% confidence interval between E-DII score and food groups' intake by multiple regression analyses in Japanese men and women aged 40–69 years.

	**Men**		**Women**	
	**Standardized beta coefficient^a^**	**(95% CI)**	**Standardized beta coefficient^a^**	**(95% CI)**
Cereals (g/day)	−0.056	(−0.076, −0.036)	−0.112	(−0.144, −0.079)
Potatoes and starch (g/day)	−0.075	(−0.091, −0.058)	−0.137	(−0.154, −0.119)
Sugar and sweetener (g/day)	0.023	(0.011, 0.035)	0.042	(0.023, 0.061)
Pulses (g/day)	−0.240	(−0.255, −0.224)	−0.227	(−0.243, −0.210)
Nuts and seeds (g/day)	−0.079	(−0.092, −0.065)	−0.091	(−0.108, −0.074)
Vegetables (g/day)	−0.386	(−0.404, −0.369)	−0.394	(−0.414, −0.373)
Fruits (g/day)	−0.194	(−0.210, −0.179)	−0.220	(−0.239, −0.202)
Mushroom (g/day)	−0.110	(−0.131, −0.090)	−0.060	(−0.076, −0.044)
Algae (g/day)	−0.079	(−0.095, −0.063)	−0.089	(−0.106, −0.071)
Fish and Shellfish (g/day)	−0.241	(−0.254, −0.227)	−0.291	(−0.310, −0.273)
Meat (g/day)	0.020	(0.004, 0.035)	−0.003	(−0.027, 0.021)
Egg (g/day)	−0.030	(−0.043, −0.017)	−0.042	(−0.059, −0.025)
Milk (g/day)	−0.042	(−0.058, −0.025)	−0.050	(−0.073, −0.027)
Fats and oil (g/day)	−0.087	(−0.104, −0.070)	−0.055	(−0.075, −0.034)
Confectioneries (g/day)	0.028	(0.011,0.045)	−0.009	(−0.028, 0.010)
Alcoholic beverages (g/day)	−0.085	(−0.102, −0.068)	−0.100	(−0.134, −0.067)
Non-alcoholic beverages (g/day)	−0.181	(−0.194, −0.167)	−0.188	(−0.205, −0.172)
Seasoning (g/day)	−0.087	(−0.100, −0.075)	−0.103	(−0.122, −0.084)

*Food groups' intake adjusted by energy intake using the residual method*.

a *Multiple regression analysis is performed with energy-adjusted dietary inflammatory index value (continuous) as the dependent variable and energy-adjusted food groups' intake as the independent variable*.

*CI, confidence interval*.

### Association Between E-DII Score and hs-CRP Concentration

Table 3 shows the mean of E-DII score and the geometric mean of hs-CRP concentration according to the E-DII quartiles. The mean E-DII score increased from −2.04 to 2.85, and the geometric mean of hs-CRP concentration increased from 0.56 mg/L to 0.67 mg/L in men, according to the E-DII quartiles. For women, the mean E-DII score increased from −3.93 to 1.88, but the geometric mean of hs-CRP concentration remained unchanged across E-DII quartiles. A significant positive association was observed between E-DII quartiles and hs-CRP concentration in men (partial regression coefficient = 0.064, *P*_trend_ < 0.01) but not in women (partial regression coefficient = 0.012, *P*_trend_ = 0.44). This means that for every increase in E-DII quartiles in men, there was a 6.6% (e^0.064^ = 1.066) increase in the geometric mean concentration of hs-CRP.

**Table 3:** Adjusted geometric mean and 95% confidence interval of high-sensitivity C-reactive protein (hs-CRP) concentration in serum (mg/L) according to quartile of energy-adjusted dietary inflammatory index (E-DII)^a^.

	***n***	**E-DII**	**hs-CRP**	**Partial regression coefficient**	***P*_trend_**
		**Mean ± SD**	**GM (95% CI)^b^, ^c^**		
**Men**					
Q1	956	−2.04 ± 1.17	0.56 (0.53, 0.59)	0.064	<0.01
Q2	956	0.20 ± 0.42	0.57 (0.54, 0.60)		
Q3	956	1.46 ± 0.34	0.62 (0.59, 0.66)		
Q4	957	2.85 ±0.57	0.67 (0.63, 0.71)		
**Women**					
Q1	662	−3.93 ± 0.83	0.46 (0.43, 0.50)	0.012	0.44
Q2	662	−1.83 ± 0.48	0.45 (0.41, 0.48)		
Q3	662	−0.16 ± 0.46	0.48 (0.44, 0.51)		
Q4	663	1.88 ± 0.93	0.47 (0.44, 0.51)		

a *E-DII is calculated from dietary intake converted per 1000 kcal*.

b *The quartile values of E-DII were entered as independent variables, and hs-CRP values were entered as dependent variables. Adjusted for age, body mass index (BMI), physical activity (MET-h/d), smoking status, and regular prescription medicine use*.

c *Geometric mean is calculated by back transforming the arithmetic mean of the log-transformed values*.

*CI, confidence interval; GM, geometric mean; Q, quartile; SD, standard deviation*.

In the sensitivity analysis stratified by age, although a positive association was not observed in participants aged 40–49 years (*P*_trend_ = 0.31), significant positive associations were observed in those aged 50–59 years (*P*_trend_ = 0.01) and 60–69 years (*P*_trend_ < 0.01) in men. Upon stratification by BMI, positive associations were consistently observed in underweight (14 kg/m^2^ ≤ BMI < 18.5 kg/m^2^, *P*_trend_ = 0.01), lower normal-weight (18.5 kg/m^2^ ≤ BMI < 22 kg/m^2^, *P*_trend_ = 0.01), higher normal-weight (22 kg/m^2^ ≤ BMI <25 kg/m^2^, *P*_trend_ < 0.01), and overweight and obese (25 kg/m^2^ ≤ BMI < 40 kg/m^2^, *P*_trend_ = 0.08) men. Even when stratified by medication status, a significant association was observed in men (non-users, *P*_trend_ < 0.01; users, *P*_trend_ < 0.01). In contrast, no significant associations were observed between E-DII quartiles and hs-CRP concentration, stratified by age and BMI, in women. Similarly, when stratified by medication status in women, no association was observed in users; however, there was a significant positive association for women non-prescription-drug users (non-users, *P*_trend_ = 0.034; users, *P*_trend_ = 0.267, see the Supplementary Table 2).

Table 4 shows the association between E-DII quartiles and higher hs-CRP (>3 mg/L) concentration. Men in the highest quartile of E-DII had 72% higher odds of having CRP >3 mg/L than men in the lowest quartile of E-DII did [OR: 1.72, 95% confidence interval (CI): 1.10–2.67, *P*_trend_ = 0.03]. In contrast, no significant association was observed among women (OR for the highest vs. lowest: 0.92, 95% CI: 0.47–1.82, *P*_trend_ = 0.96).

**Table 4:** Adjusted odds ratios and 95% confidence interval for the association between energy-adjusted dietary inflammatory index (E-DII) quartile^a^ and >3 mg/L of high-sensitivity C-reactive protein (hs-CRP).

	**Men**			**Women**		
	***n* (%)**	**OR (95% CI)^b^**	***P*_trend_**	***n* (%)**	**OR (95% CI)^b^**	***P*_trend_**
Q1	38 (4.0)	1	0.03	22 (3.3)	1	0.96
Q2	45 (4.7)	1.21 (0.78, 1.89)		17 (2.6)	0.74 (0.38, 1.43)	
Q3	40 (4.2)	1.15 (0.72, 1.83)		21 (3.2)	0.94 (0.50, 1.78)	
Q4	57 (6.0)	1.72 (1.10, 2.67)		21 (3.2)	0.92 (0.47, 1.82)	

a *E-DII is calculated from dietary intake converted per 1,000 kcal*.

b *Adjusted for age, body mass index (BMI), physical activity (MET-h/d), smoking status, regular prescription medicine use*.

*Number of participants who had higher hs-CRP values (>3 mg/L) is expressed as n (%)*.

*CI, confidence interval; CRP, C-reactive protein; E-DII, energy-adjusted dietary inflammatory index; OR, odds ratio; Q, quartile*.

## Discussion

The DII was developed to evaluate the inflammatory potential of people's diets. The present study was conducted to validate the E-DII with a hs-CRP concentration in a large number of Japanese participants. The findings of our study suggest that there are significant positive associations in all men and only in women who are prescription drug non-users.

The positive association in Japanese men observed in the present study is consistent with the results of previous studies from Western countries (15–17) and Japan (18, 23). The dietary habits of the Japanese are different from those of the Western people, and the CRP concentrations of the Japanese (0.6 mg/L in the men of the present study) are considerably lower than those of Westerners [typically, they are approximately 2–3 mg/L (15, 17)]. Therefore, the positive association observed in Japanese men in the present study suggests that DII may apply to a diverse male population with considerably different dietary habits and a different range of inflammatory status.

As in previous studies, inconsistent results were observed between Japanese men and women in the present study. We could not detect any associations between E-DII scores and hs-CRP concentrations across women in this study. However, we did observe a positive association when we restricted the analyses to non-users of prescription medicine. The mean hs-CRP concentration was significantly lower in women than in men. In addition, the proportion of participants with hs-CRP concentration >3.0 mg/L was significantly lower in women than in men. Therefore, owing to the low concentration of hs-CRP in Japanese women in this study, detection of the association between E-DII score and hs-CRP concentration may be difficult. This may have also been responsible for the limited positive association between DII and hs-CRP concentration in non-users of prescription drugs among women. In this study, we excluded from the analysis subjects with a history of cancer, stroke, or myocardial infarction, which could be confounding. However, approximately half of the subjects were regular users of some prescription medication. It has been reported that hs-CRP concentrations are higher in hypertension patients than in healthy subjects (33) and that hs-CRP concentrations are higher in diabetes patients with high HbA1c concentrations (34). Considering these previous studies, hs-CRP concentrations may be high in populations with some disease taking prescribed medication. In fact, in the current study, prescription drug users of both sexes had higher hs-CRP concentrations than non-users did. The difference in the percentage of people with high hs-CRP concentration was particularly apparent in women (see Supplementary Table 1). Therefore, we suggest that in women, the association between DII and hs-CRP concentration may have been more clearly demonstrated in non-drug users only. Of note, studies in populations with low hs-CRP concentrations are limited; therefore, further studies are needed to determine the utility of DII in populations with low CRP concentrations, such as Japanese women.

Another underlying reason for the null finding among Japanese women may be the differences in dietary habits between men and women. The women's diets had significantly lower inflammatory potential, indicated by the negative value of mean E-DII score, than those of men, who showed a positive value of mean E-DII score. This is in line with the results of our previous study, wherein the E-DII scores of women were much lower than those of men, and no associations were observed in women in that study either (23). Further, similar results have been reported in a previous study conducted in postmenopausal women from USA, wherein mean E-DII score was a negative value (−0.62 ± 2.69), and a null association was observed between E-DII score and hs-CRP concentration despite having comparatively higher hs-CRP concentration (mean 1.36 mg/L) (35). Taking into consideration the aforementioned reports, it is possible that if the target population eats a predominantly anti-inflammatory diet, the association between DII score and hs-CRP concentration may be difficult to detect.

The dietary patterns that constitute E-DII may vary among populations. Among men with an association between E-DII and inflammatory markers in this study, the food groups positively associated with E-DII were meat and confectioneries. Similarly, in NIPPON DATA, wherein the association with inflammatory markers was confirmed, meat and confectioneries, as well as cereals and fats, were positively associated with E-DII (18). Contrastingly, in the male population of the JPHC-FFQ validation study, meat and confectioneries were not associated with E-DII. The higher the E-DII, the lower the intake of potatoes, legumes, vegetables, and seaweed (23). The study period of the current study (2009–2013) is similar to that of the NIPPON DATA2010 study (18), but it differs from that of the JPHC-FFQ validation study (1990–1993) (23). Thus, the differences in the dietary habits due to cohort effects may contribute to the differences in the dietary patterns involved in E-DII. Further studies in various populations are needed to determine the types of dietary patterns involved.

This study has several limitations. First, the participants of the present study were individuals who had voluntarily undergone cancer screening. These participants, especially women, may be particularly health conscious as indicated by their smoking rate, which was considerably lower than that observed in the National Health and Nutrition Survey in Japan: approximately 15% men and 5% women in the current study compared with 30% men and 10% women in the national survey (36). This selection bias might have contributed to the null association between E-DII score and hs-CRP concentration, observed in women. Second, because this is a cross-sectional study, we could not account for the temporality requirement for assessing causality. Despite these limitations, the present study is a relatively large-scale study, and the results indicated gender differences in E-DII validity and reaffirmed validity in Japanese men. Thus, this justifies the use of E-DII in Japanese men.

To conclude, we conducted a validation study of E-DII using hs-CRP concentration in Japanese men and women and observed a positive association between E-DII scores and hs-CRP concentration in Japanese men, even after adjusting for age, BMI, smoking status, regular prescription medicine use, and physical activity. This indicates the utility of the E-DII in Japanese men who have different dietary habits and considerably lower-grade inflammation status than those of the Western population. However, we could not detect any association between E-DII scores and hs-CRP concentration in Japanese women, except for prescription medication non-users. Therefore, further studies are needed to clarify the utility of the E-DII in Japanese women.

## Data Availability Statement

We cannot publicly provide individual data due to participant privacy, according to ethical guidelines in Japan. Additionally, the informed consent we obtained does not include a provision for publicly sharing data. Qualifying researchers may apply to access a minimal dataset by contacting Dr. Shoichiro Tsugane, Principal Investigator, Epidemiology and Prevention group, Center for Public Health Sciences, National Cancer Center, Tokyo, Japan, at stsugane@ncc.go.jp. Or, please contact the Office of the Research Center for Cancer Prevention and Screening Program at tyamaji@ncc.go.jp.

## Ethics Statement

This study was approved by the Institutional Review Board of the National Cancer Center, Tokyo, Japan (approval number G15-01 and 2016-165). The study aims and protocols were explained to all participants, and each participant provided written informed consent before enrolment in the study.

## Author Contributions

AK, NSa, MIw, JI, and MIn designed the study. ST, JI, NSa, MIw, and MIn arranged the field survey. AK, NSa, MIw, and TY contributed to the blood analysis. NSh and JH conducted DII calculation and provided critical input to the manuscript. AK performed statistical analysis, interpreted the results, and drafted the manuscript. All authors contributed to the article and approved the submitted version.

## Conflict of Interest

JH owns a controlling interest in Connecting Health Innovations LLC (CHI), a company planning to license the right to his invention of the dietary inflammatory index (DII), from the University of South Carolina, to develop computer and smartphone applications for patient counseling and dietary intervention in the clinical setting. NSh is an employee of CHI. The subject matter of this paper will have no direct bearing on the work of CHI, nor has any CHI-related activity exerted any influence on this project. MI was the beneficiary of a financial contribution from the AXA Research Fund as a chair holder of the AXA Department of Health and Human Security at The University of Tokyo. The AXA Research Fund had no role in the design, data collection, analysis, interpretation, manuscript drafting, or in the decision to submit the manuscript for publication. The remaining authors declare that the research was conducted in the absence of any commercial or financial relationships that could be construed as a potential conflict of interest.

## Abbreviations

BMI: body mass index
CI: confidence interval
CV: coefficient of variation
DII: dietary inflammatory index
E-DII: energy-adjusted dietary inflammatory index
FFQ: food frequency questionnaire
hs-CRP: high-sensitivity C-reactive protein
MET: metabolic equivalent
OR: odds ratio
SD: standard deviation.
