# Supplementary material for: Dietary Inflammatory Index Is Associated With Inflammation in Japanese Men
Source: Front Nutr. 2021 Apr 9;8:604296. doi: 10.3389/fnut.2021.604296 (PMC8062774; doi:10.3389/fnut.2021.604296)
Supplement: Supplementary file 1 [file Data_Sheet_1.docx]

| Supplementary Table 1. Comparisons of mean and median values of characteristics between prescription drug non-users and users, between men and women of non-users, and between men and women of users. | | | | | | | | | | | | | | | | | | | | | | | | | | | | |
| --- | --- | --- | --- | --- | --- | --- | --- | --- | --- | --- | --- | --- | --- | --- | --- | --- | --- | --- | --- | --- | --- | --- | --- | --- | --- | --- | --- | --- |
|  | **Men** | | | | | | | | | | |  |  | **Women** | | | | | | | | | | |  |  |  |  |
|  | **Non-users** | | | | |  | **Users** | | | | | p-value |  | **Non-users** | | | | |  | **Users** | | | | | p-value |  |  |  |
|  | **(n=1986)** | | | | |  | **(n=1839)** | | | | |  |  | **(n=1464)** | | | | |  | **(n=1185)** | | | | |  |  | **between non-users ^c^** | **between users ^d^** |
|  | Mean ± SD | | | Median (interquartile range) | |  | Mean ± SD | | | Median (interquartile range) | |  |  | Mean ± SD | | | Median (interquartile range) | |  | Mean ± SD | | | Median (interquartile range) | |  |  | p-value | p-value |
| Age (years) | 54.7 | ± | 8.4 | 55 | (49, 62) |  | 58.7 | ± | 7.60 | 60 | (54, 65) | <0.001 |  | 54.9 | ± | 8.2 | 55 | (48, 62) |  | 58.6 | ± | 7.6 | 60 | (53, 65) | <0.001 |  | 0.757 | 0.703 |
| Height (cm) | 169.9 | ± | 5.9 | 170 | (166, 174) |  | 169.0 | ± | 5.84 | 169 | (165, 173) | <0.001 |  | 156.8 | ± | 5.5 | 157 | (153, 161) |  | 156.0 | ± | 5.5 | 156 | (152, 160) | <0.001 |  | <0.001 | <0.001 |
| Weight (kg) | 68.0 | ± | 9.2 | 67 | (62, 74) |  | 69.3 | ± | 9.72 | 68 | (63, 75) | <0.001 |  | 53.3 | ± | 7.6 | 52 | (48, 57) |  | 54.2 | ± | 8.6 | 53 | (49, 58) | 0.011 |  | <0.001 | <0.001 |
| BMI (kg/m2) | 23.5 | ± | 2.8 | 23 | (22, 25) |  | 24.2 | ± | 2.93 | 24 | (22, 26) | <0.001 |  | 21.7 | ± | 2.9 | 21 | (20, 23) |  | 22.3 | ± | 3.3 | 22 | (20, 24) | <0.001 |  | <0.001 | <0.001 |
| Smoking status, % |  |  |  |  |  |  |  |  |  |  |  |  |  |  |  |  |  |  |  |  |  |  |  |  |  |  |  |  |
| Current | 17.2 | | |  |  |  | 13.5 | | |  |  | <0.001 |  | 5.7 | | |  |  |  | 5.1 | | |  |  | 0.798 |  | <0.001 | <0.001 |
| Former | 47.1 | | |  |  |  | 57.5 | | |  |  |  |  | 15.2 | | |  |  |  | 15.2 | | |  |  |  |  |  |  |
| Never | 35.7 | | |  |  |  | 28.9 | | |  |  |  |  | 79.0 | | |  |  |  | 79.7 | | |  |  |  |  |  |  |
| Physical activity (MET-h/d) | 36.6 | ± | 3.3 | 36 | (35, 38) |  | 36.5 | ± | 3.1 | 36 | (35, 38) | 0.811 |  | 38.0 | ± | 3.8 | 37 | (36, 39) |  | 37.9 | ± | 3.6 | 37 | (36, 39) | 0.269 |  | <0.001 | <0.001 |
| E-DII, /1000 kcal ^a^ | 0.83 | ± | 1.90 | 1.10 | (-0.31, 2.26) |  | 0.39 | ± | 1.95 | 0.61 | (-0.83, 1.78) | <0.001 |  | -0.83 | ± | 2.27 | -0.69 | (-2.44, 0.84) |  | -1.23 | ± | 2.22 | -1.30 | (-2.89, 0.41) | <0.001 |  | <0.001 | <0.001 |
| Crude hs-CRP, mg/L | 0.86 | ± | 1.10 | 0.5 | (0.3, 1.0) |  | 0.96 | ± | 1.19 | 0.6 | (0.3, 1.1) | <0.001 |  | 0.66 | ± | 0.95 | 0.4 | (0.2, 0.7) |  | 0.81 | ± | 1.14 | 0.4 | (0.2, 0.9) | <0.001 |  | <0.001 | <0.001 |
| hs-CRP, mg/L ^b^ | 0.55 | ± | 1.21 | - | |  | 0.62 | ± | 1.20 | - | | <0.001 |  | 0.40 | ± | 1.21 | - | |  | 0.48 | ± | 1.25 | - | | <0.001 |  | <0.001 | <0.001 |
| >3 mg/L of hs-CRP, % | 4.2 | | |  |  |  | 5.3 | | |  |  | 0.110 |  | 2.5 | | |  |  |  | 3.8 | | |  |  | 0.047 |  | 0.006 | 0.061 |
| Chi-square test and Mann-Whitney U test are used for statistical analyses. BMI: Body mass index; E-DII: Energy-adjusted dietary inflammatory index; hs-CRP: high-sensitive C-reactive protein; MET: Metabolic equivalent. | | | | | | | | | | | | | | | | | | | | | | | | | | | | |
| ^a^ E-DII is calculated from dietary intake converted per 1000 kcal. | | | | | | | | | | | | | | | | | | | | | | | | | | | | |
| ^b^ Geometric mean and coefficient of variation were presented for log-transformed inflammatory biomarkers. Coefficient of variation is calculated using the formula: CV= (eSD-1)1/2. | | | | | | | | | | | | | | | | | | | | | | | | | | | | |
| ^c^ Comparing between men and women among prescription drug non-users. | | | | | | | | | | | | | | | | | | | | | | | | | | | | |
| ^d^ Comparing between men and women among prescription users. | | | | | | | | | | | | | | | | | | | | | | | | | | | | |

| Supplementary Table 2. Adjusted geometric mean and 95% confidence interval of high-sensitivity C-reactive protein (hs-CRP) concentration in serum (mg/L) according to quartile of energy-adjusted dietary inflammatory index (E-DII)^a^ stratified by the status of prescription drug use | | | | | | | | | | | |
| --- | --- | --- | --- | --- | --- | --- | --- | --- | --- | --- | --- |
|  | **Men** | | | | |  | **Women** | | | | |
|  | hs-CRP | | | Partial regression coefficient |  |  | hs-CRP | | | Partial regression coefficient |  |
|  | GM (95% CI)^b, c^ | | |  | *P*_trend_ |  | GM (95% CI)^b, c^ | | |  | *P*_trend_ |
| Prescription drug non-users | | | | | | | | | | | |
| Q1 | 0.51 | ( 0.47, | 0.55 ) | 0.075 | <0.01 |  | 0.39 | ( 0.35, | 0.44 ) | 0.045 | 0.034 |
| Q2 | 0.53 | ( 0.49, | 0.57 ) |  |  |  | 0.40 | ( 0.36, | 0.45 ) |  |  |
| Q3 | 0.59 | ( 0.55, | 0.64 ) |  |  |  | 0.42 | ( 0.38, | 0.46 ) |  |  |
| Q4 | 0.63 | ( 0.58, | 0.67 ) |  |  |  | 0.45 | ( 0.41, | 0.50 ) |  |  |
|  |  |  |  |  |  |  |  |  |  |  |  |
| Prescription drug users | | | | | | | | | | | |
| Q1 | 0.61 | ( 0.56, | 0.66 ) | 0.052 | <0.01 |  | 0.55 | ( 0.49, | 0.62 ) | -0.027 | 0.267 |
| Q2 | 0.61 | ( 0.56, | 0.66 ) |  |  |  | 0.50 | ( 0.45, | 0.57 ) |  |  |
| Q3 | 0.65 | ( 0.60, | 0.71 ) |  |  |  | 0.55 | ( 0.49, | 0.62 ) |  |  |
| Q4 | 0.72 | ( 0.66, | 0.79 ) |  |  |  | 0.49 | ( 0.43, | 0.56 ) |  |  |
| ^a^ E-DII is calculated from dietary intake converted per 1000 kcal. | | | | | | | | | | | |
| ^b^ The quartile values of E-DII were entered as independent variables, and hs-CRP was entered as a dependent variable. Adjusted for age, body mass index (BMI), physical activity (MET-h/d), smoking status, regular prescription medicine use | | | | | | | | | | | |
| ^c^ Geometric mean is calculated by back transforming the arithmetic mean of the log-transformed values. | | | | | | | | | | | |
| CI: Confidence interval; GM: Geometric mean; Q: Quartile; SD: Standard deviation | | | | | | | | | | | |
